# Collectanea Medica: Consisting of Anecdotes, Facts, Extracts, Illustrations, &c.

**Published:** 1823-08

**Authors:** 


### COLLECTANEA MEDICA:

CONSISTING OF

ANECDOTES, FACTS, EXTRACTS, ILLUSTRATIONS, &c.

*Relating to the History or the Art of Medicine, and the Collateral Sciences.*

Floriferis, ut apes, in saltibus omnia libant,
Omnia nos, itidem, depascimur aurea dicta.

### ART. I.—*Extracts from "Medical Jurisprudence."* By J. A. PARIS, M.D. &c. and J. S. M. FONBLANQUE, Esq.

### *Of Medical Evidence generally.*

As physicians, surgeons, and others conversant in medicine and chemistry, are constantly called upon to give testimony in courts of justice, it is necessary for us to enter upon this subject of the law of evidence, so far as it immediately affects the medical witness. It is proper that he should understand when he is bound to appear, and on what terms; and it may be useful for him to be prepared, by some previous knowledge of the usual course of examination, for the difficulties and objections which may arise in the progress of it. A scientific witness, fully acquainted with the subject in dispute, and, by his particular knowledge, well qualified to inform the Court on the most important points, is too frequently rendered miserable in himself, and absolutely ineffective to the ends of justice, by the diffidence which a man of real acquirement generally feels, when impressed at once with the novelty of his situation, a sense of the importance of the duty which he is about to perform, and a consciousness that the truths which he is about to utter may be obscured, suppressed, or perverted, by technicalities for which he is unprepared with any defence. We do not mean to arraign the present forms of examination in general, when we assert that some abuse in practice too frequently places the witness in as painful a situation as if he were himself a criminal.

Some knowledge of the law of evidence is the best security against this inconvenience: we propose, therefore, to lay down a few general rules on the points most likely to occur, and to refer our readers for more particular information to those works which expressly or incidentally treat on this subject.

It is necessary, in the first place, to consider how the attendance of witnesses is to be compelled by process, under what terms they must appear, their liabilities if they fail to appear, and their duties when in court.

The writ of subpœna *ad testificandum* is the ordinary process of the Courts for compelling the attendance of witnesses: by this the intended witness is required to appear at the trial at a fixed time and place, to testify what he knows in the cause, under the penalty of 100*l*. to be forfeited to the King.

Four witnesses may be included in one subpœna; but a ticket containing the substance of the writ (which is to be shown at the same time,) is as effectual service as the writ itself. The service must be upon the witness in person, and within reasonable time before the trial, respect being always had to the residence and circumstances of the party.

As attendance is more burthensome on a professional man than on others, so also it is more frequently called for. Men, in general, can only be summoned as witnesses when they have, or are reasonably supposed to have, cognisance of the particular facts in question; and he may therefore deem himself peculiarly unfortunate or imprudent who is often present at such scenes as give rise to criminal instigation. But the medical practitioner, in addition to his liability of being called in for his assistance, and so becoming acquainted with facts, may also be summoned on matters of opinion; those, therefore, who stand highest in public estimation as men of science and research, will be most frequently burthened with the execution of painful and unprofitable duties. We do not believe that they will shrink from the performance of them when necessary, but we may express a hope that they may be rendered as little burthensome as their nature will allow.

Great difficulties must always arise in the examination of a medical or chemical witness, where the examining party is uninformed, or at least very partially acquainted, with the science in question; for it is next to impossible for counsel so to frame their examination of a scientific witness as to elicit the whole truth, unless they are, by previously acquired knowledge, acquainted with the bearings of each answer upon the case which they are maintaining; and, though there are a few instances of persons of such superior talent that they can collect from the mere information of their briefs so much knowledge as will enable them to perform this duty with credit to themselves and satisfaction to their clients and the public, yet such instances are rare; and even those most gifted will admit that there is a most material difference between examining a witness on matters of fact, of which all persons who have applied themselves to the laws and nature of evidence may be competent judges, and the examination of abstract opinions and speculations of philosophy or physics, where the examiner can as little follow the reasoning of a witness, as if he spoke some foreign and unknown language. For it is impossible, within the compass of any ordinary *viva voce* examination, to elicit all the points on which explanation may be necessary, or to remove all the doubts which may give occasion to future controversy: hence questions of this kind are seldom determined at the first hearing, but are repeatedly brought before the courts in the form of new trials. The cases of *Severn, King, and Co.* against several Fire-Insurance offices, which in part suggested the undertaking of the present work, may serve as an elucidation of this point. The causes were conducted by professional men of the first eminence; the judge who presided well known for his love of science, and from having attained more knowledge in several branches of natural philosophy than can usually be acquired by those whose time is engrossed by severer studies; the witnesses were among the best chemists of the day; yet the question (simple as it might at first appear,) whether oil or sugar, at certain temperatures, and under certain circumstances, should be considered the more inflammable substance, occupied three days on the first, and six days on the second, trial. Notwithstanding which, a third trial took place, involving the same question, and controversial pamphlets were published on both sides, on the nature and supposed contradictions of the evidence.

It has been supposed that medical practitioners may avail themselves of the privilege enjoyed by legal advisers, and that they are not bound to divulge the secrets of their patients, reposed in them in the course of professional confidence. Undoubtedly this confidence ought not to be violated on any ordinary occasion; but, when the ends of justice absolutely require the disclosure, there is no doubt that the medical witness is not only bound, but compellable, to give evidence; ever bearing in mind that the examination should not be carried further than may be relevant to the point in question: of this the Court will judge, and protect the witness accordingly.

As to the mode in which a medical witness should deliver his evidence, very different advice appears to have been given by different authorities: while some, impatient of delay, and dreading the arts of examination, recommend their pupils or readers to open at once all the stores of their reasoning and information, others, fearing the effect which cross-examination may have on nervous or embarrassed witnesses, advise that no more shall be disclosed than categorically meets the question of the counsel: and to this we incline, with this difference, that, as we should deem too costive a retention of the truth as blamable as the flow of garrulity with which we have sometimes seen a court overwhelmed, we recommend the witness to steer a middle course,—first answering patiently, distinctly, and tersely, the questions put by the counsel on both sides, the Court, and the jury; and, if none of these elicit the whole truth, and any material point remains to be disclosed, the presiding judge will always admit, and gratefully receive, the additions or explanations which may be necessary to the ends of justice.

The witness is next to consider what is, and what is not, evidence. We cannot follow this subject in all its bearings, nor indeed is it here necessary: a few points must, however, be remembered; and first of notes. These, if taken upon the spot or immediately after a transaction, may be used by the witness to refresh his memory; and, as to dates, numbers, or quantities, it is generally expedient to have them. The notes should be original, not copies. If there be any point in them which the witness does not recollect, except that he finds it there, such point is not evidence; for the notes are only to assist recollection, not to convey information.

The witness must relate only that which he himself has seen or observed: that which he has heard from others is not evidence, as coming from him; except, indeed, where some expressions or declarations of the parties concerned have become a part of the *res gesta.* But the declarations of a dying man are evidence when related by a third person on oath, though the party making them was not sworn; for the law presumes that the solemnity of the occasion may dispense with the form, and that a man, trembling on the brink of eternity, will never risk salvation by falsehood. To give this weight to a declaration, it is necessary that the party should believe himself to be dying. Mr. Justice Bailey is reported to have said, that the party must be satisfied that recovery was impossible. We think the reporter must have been mistaken; for such a rule would exclude all such declarations: hope is the latest faculty of the human mind. "I am better," has not unfrequently been the last articulation of expiring nature.

How far, and in what cases, opinion is evidence, is next to be considered. In ordinary matters where, from a statement of facts, the jury, in the exercise of sound and ordinary understanding, are capable of arriving at a just conclusion, the opinion of a witness is neither requisite nor admissible; but in matters of science it is otherwise, provided that he backs his opinion by such reason as may be satisfactory to the understanding of his hearers: and this is the principal qualification of a medical witness, that he make himself *intelligible to ordinary comprehensions.*

No man is bound to give any evidence by which he may render himself liable to any criminal prosecution. At the Old-Bailey Sessions, in June 1821, Mr. George Patmore was tried for the murder of John Scott, in a duel. Mr. Pettigrew, a surgeon, was the first witness called.

*Mr. Justice Bailey.*—Mr. Pettigrew, I think it necessary to give you this caution: if you think the evidence which you are about to give likely to expose you to a criminal prosecution, you are not bound to give it.

*Mr. Pettigrew.*—My Lord, I am not competent to form any opinion of my legal guilt; I have not taken the part of principal or second. The part which I have taken was merely to exercise my professional duty: in that I do not think there is any moral guilt.

*Mr. Justice Bailey.*—If you went (knowing a duel was to take place) for the purpose of giving surgical assistance, I apprehend that you are liable to a criminal prosecution.

*Mr. Pettigrew.*—Then, my Lord, I must decline answering any questions.

*Mr. Justice Bailey.*—I recollect having seen a surgeon of eminence tried in this court, on a similar occasion.

Neither Mr. Pettigrew nor his assistant were examined.

Dr. Darling, who had attended the deceased after he had received his wound, deposed that he heard Mr. Scott, on his death-bed, say—

*Mr. Justice Bailey.*—Did Mr. Scott at that time think himself in danger: did he give up all hopes of recovery.

*Dr. Darling.*—No. To the last he entertained hopes of recovery.

*Mr. Justice Bailey.*—The declaration made by a dying man cannot be received as evidence, unless the party at the time of making it were satisfied that recovery was impossible.

We have before noticed the limitation with which we believe this supposed rule must be taken.

With the exception of dying declarations, all evidence in criminal matters must be upon oath; therefore the affirmation of a quaker cannot be received on a coroner's inquest. In the too-celebrated case of the Oldham inquest on the body of John Lees, Mr. Earnshaw, a quaker surgeon, who had attended the deceased, though much urged, refused to be sworn, and his testimony was consequently rejected.* A paper was subsequently delivered to the jury, containing the matter of his observation: this was very properly resented by the coroner, as an illegal attempt to influence the jury, who by their oaths were bound to admit no information which wanted that legal sanction. While we were writing this article, we were surprised to find that a coroner for the county of Surrey had permitted the letter of a physician to be read to the jury, as evidence that a person deceased was of unsound mind; and on this evidence (for we can scarcely suppose that the servant's deposition to rheumatic head-aches was allowed to weigh,) a verdict of insanity was returned.

---

* Baptist or quaker surgeons should therefore, in cases likely to come before the criminal tribunals, take care to have persons associated with them who may supply their places in court. We do not urge them to be sworn, as we should place less reliance on an oath taken in breach of conscientious scruples, than on the affirmation which is rejected in obedience to the forms of law.

As a quaker, if living, could not be heard as a witness in a criminal case, query his declarations when dying, does the solemnity of the occasion dispense with the form of an oath?

